# The Lysyl Oxidase Inhibitor, β-Aminopropionitrile, Diminishes the Metastatic Colonization Potential of Circulating Breast Cancer Cells

**DOI:** 10.1371/journal.pone.0005620

**Published:** 2009-05-19

**Authors:** Alla Bondareva, Charlene M. Downey, Fabio Ayres, Wei Liu, Steven K. Boyd, Benedikt Hallgrimsson, Frank R. Jirik

**Affiliations:** 1 Department of Biochemistry and Molecular Biology, University of Calgary, Calgary, Alberta, Canada; 2 Department of Mechanical and Manufacturing Engineering, University of Calgary, Calgary, Alberta, Canada; 3 Department of Cell Biology and Anatomy, University of Calgary, Calgary, Alberta, Canada; 4 The McCaig Institute for Bone and Joint Health, University of Calgary, Calgary, Alberta, Canada; Uppsala University, Sweden

## Abstract

Lysyl oxidase (LOX), an extracellular matrix remodeling enzyme, appears to have a role in promoting breast cancer cell motility and invasiveness. In addition, increased LOX expression has been correlated with decreases in both metastases-free, and overall survival in breast cancer patients. With this background, we studied the ability of β-aminopropionitrile (BAPN), an irreversible inhibitor of LOX, to regulate the metastatic colonization potential of the human breast cancer cell line, MDA-MB-231. BAPN was administered daily to mice starting either 1 day prior, on the same day as, or 7 days after intracardiac injection of luciferase expressing MDA-MB-231-Luc2 cells. Development of metastases was monitored by *in vivo* bioluminescence imaging, and tumor-induced osteolysis was assessed by micro-computed tomography (μCT). We found that BAPN administration was able to reduce the frequency of metastases. Thus, when BAPN treatment was initiated the day before, or on the same day as the intra-cardiac injection of tumor cells, the number of metastases was decreased by 44%, and 27%, and whole-body photon emission rates (reflective of total tumor burden) were diminished by 78%, and 45%, respectively. In contrast, BAPN had no effect on the growth of established metastases. Our findings suggest that LOX activity is required during extravasation and/or initial tissue colonization by circulating MDA-MB-231 cells, lending support to the idea that LOX inhibition might be useful in metastasis prevention.

## Introduction

Despite advances in early detection and improvements in the treatment of breast cancer, mortality remains relatively high and is invariably associated with the development of metastases. Indeed, approximately one third of patients that are treated for localized breast cancer will eventually develop recurrence at distant sites [1]. Once metastases are present, average survival decreases to approximately 24 months [1], [2]. The metastatic process is thought to involve multiple steps, including loss of cell-to-cell and cell-to-basement membrane adhesion, epithelial-mesenchymal transition, and increased cell motility. After migrating though the connective tissue and the lymphatic or vascular endothelium, cells must be able to avoid anoikis, adhere to the microcapillary endothelium, and then extravasate before being able to generate metastatic colonies within favorable microenvironmental niches [3], [4], [5], [6]. Many of these steps involve dynamic interactions between tumor cells, stromal cells, and the extracellular matrix (ECM) [7]. In keeping with the latter, expression microarray data has revealed that genes encoding ECM remodeling proteins are frequently over-expressed within the tumor stroma, and that dysregulation of ECM-relevant genes is predictive of metastasis in both mouse and human mammary cancers [8], [9]. Within this context, lysyl oxidase (LOX) is thought to play an important role in modulating tumor behavior.

LOX, an extracellular matrix-remodeling enzyme, is required for the oxidative deamination of lysine residues in collagen and elastin molecules that is required for fiber cross-linking; hence, LOX controls both the structure and the tensile strength of ECM, and thus acts to preserve tissue integrity [10]. The activities of LOX and other members of LOX family (that include both extracellular and intracellular isoforms) are complex, having been shown to regulate events such as chromatin compaction [11], gene transcription [12], [13], as well as cell differentiation and tissue development [14]. The findings that LOX activity is modulated by oxygen levels [15], [16], [17], and also that LOX is able to regulate cell migration and adhesion [18], [19], have generated considerable interest in the role of LOX during tumor progression. In particular, it has been shown that hydrogen peroxide, released as a consequence of LOX mediated catalysis, triggers the phosphorylation and activation of two key signal transduction pathway activators: Src and focal adhesion kinase (FAK). FAK, for example, activates multiple intracellular signal transduction pathways that modulate actin filament formation, turnover of cellular-ECM adhesion complexes, formation of lamellipodia, and expression of matrix metalloproteinases; all processes that are involved in tumor cell invasiveness and migration [17], [18], [19], [20].

Both up- and down-regulation of LOX has been observed in different cancer cell lines and primary tumors (reviewed in [21]), however, in breast cancer, elevated LOX expression has been positively-correlated with invasiveness, as well as with reduced metastasis-free and overall survival [16], [22], [23], [24], [25]. In an elegant series of experiments, Erler and colleagues identified a role for LOX in breast cancer cells, showing that by reducing the activity of this molecule (by either chemical inhibition or RNAi) lung metastases could be greatly decreased, and liver metastases eliminated in mice bearing orthotopic MDA-MB-231 tumors [16]. However, the question remained as to whether LOX inhibition was acting (a) to prevent the migration of cells away from the primary tumor, (b) by limiting tumor cell intravasation, (c) by blocking extravasation, or (d) by decreasing the ability of the tumor cells to colonize favorable niches within distant sites. We therefore evaluated the ability of the irreversible LOX inhibitor, β-aminopropionitrile (BAPN), to regulate metastatic colonization and growth of the MDA-MB-231 breast cancer cells following their introduction into the arterial circulation of immunodeficient mice. Our findings demonstrate that BAPN significantly reduced the frequency of metastases in both soft tissue and skeletal sites, while having no effect on the growth of established metastases. Our findings suggest that LOX likely plays an important role in regulating extravasation and/or tissue colonization by circulating breast cancer cells.

## Materials and Methods

### Cell culture

The human breast cancer cell line, MDA-MB-231, was kindly provided by Dr. T. Guise (University of Virginia, Charlottesville), and were confirmed to be free of pathogenic murine viruses and *Mycoplasma spp.* by PCR testing at Charles River Laboratories (Wilmington, MA). Cells were grown in Dulbecco's modified Eagle medium (DMEM; Invitrogen, Grand Island, NY) supplemented with 10% heat-inactivated fetal bovine serum (FBS), 100 U/ml penicillin, 100 µg/ml streptomycin at 37°C in a 5% CO_2_ humidified atmosphere, and were routinely passaged every 2–3 d.

### Construction of dual-reporter plasmid vector

A 1652-bp fragment of the plasmid pGL4.13 (Promega, Madison, WI), encoding the firefly luciferase (luc2) cDNA (nt 499–2151; GenBank accession number AY738225), was PCR-amplified using *Vent* DNA-polymerase (New England Biolabs, Mississauga, ON). The primers used for the PCR-amplification contained an *Eco*R1 and *Bam*H1 site, respectively, and were: sense, 5′ – ATAGAATTCATGGAAGATGCCAAAAACATTAAG; and antisense, 5′ – TATGGATCCTAGAATTATTACACGGCGATCTTG. The fragment encoding luciferase was then subcloned into the *Eco*R1 and *Bam*H1 sites within the polylinker region of the enhanced green fluorescence protein (EGFP)-expressing pEGFP-C2 vector (Clontech, Mountain View, CA), such that the luciferase sequence was inserted in-frame and downstream of the EGFP coding region. The sequence of pEGFP-Luc2 vector was then confirmed both by restriction mapping and DNA sequencing of the plasmid.

### Transfection of breast cancer cells

MDA-MB-231 cells were stably transfected with the pEGFP-Luc2 vector using the Lipofectamine reagent (Invitrogen) according to the manufacturer's instructions. Transfectants were selected in media containing 1.2 mg/ml geneticin (Invitrogen) for 21 d, and then re-selected by fluorescence-activated cell sorting (FACS)(Flow Cytometry Core Facility, University of Calgary) to enrich for a population of cells that exhibited the highest EGFP fluorescence signal intensities. Individual clones of sorted cells were also analyzed for the expression of luciferase, and 60 clones exhibiting the highest levels of EGFP-Luc2 expression were pooled so as to maintain a level of potential variability comparable to that of the parental cells. This pooled population was then expanded, aliquoted, and stored in liquid N_2_, so that the identical cell population could be used for all subsequent *in vivo* experiments.

### Cell proliferation assay

Growth of MDA-MB-231-Luc2 and the parental MDA-MB-231 cells was compared using 3-(4,5-dimethyl-2-thiazolyl)-2,5-diphenyl-2H-tetrazolium bromide (MTT reagent; Sigma, St. Louis, MO) according to the manufacturer's instructions. Briefly, cells were seeded in 96-well plates at 1×10^3^ cells per well in 100 µl of culture medium and grown under normal conditions at 37°C in humidified atmosphere. After incubation for up to 5 d, the medium was discarded and replaced with 90 µl of fresh medium, followed by the addition of 10 µl of 5 mg/ml MTT reagent and incubation for 4 hr at 37°C for cells to develop the formazan product. Subsequently, the MTT-containing medium was aspirated, and 100 µl of DMSO (Sigma) was added to lyse the cells and to solubilize the formazan. Absorbance values of the lysates were determined on a Multiskan Ascent microplate reader (Thermo Labsystems, Helsinki, Finland), using a background wavelength of 620 nm and a detection wavelength of 550 nm.

### Luciferase expression by MDA-MB-231-Luc2 cells

Luciferase expression by MDA-MB-231 cells stably transfected with the pEGFP-Luc2 plasmid was assessed using a Xenogen IVIS Imaging System instrument (Caliper Life Sciences; Alameda, CA). Briefly, serial dilutions of 2×10^3^ to 1×10^4^ cells per well were plated into black, clear bottomed, 96-well plates (Costar; Acton, MA). *D*-luciferin (Gold Bio Technology; St. Louis, MO) was then added to a final concentration of 150 µg/ml in medium 10 min before imaging. Bioluminescence readings were analyzed with Living Image 3.0 software (Caliper Life Sciences; Alameda, CA).

### Mice

Nude-beige (NIH-III) female mice (4–5 week old) were purchased from Charles River Laboratories (St. Constant, QC). Mice were housed under viral antibody-free conditions in the biohazard area of the University of Calgary Animal Resources Center in compliance with Canadian Council of Animal Care guidelines and ethical approval from the University of Calgary Animal Care Committee.

### Experimental metastasis model and drug administration

To generate metastases, 5–6 week old female NIH-III mice were anesthetized by intraperitoneal (i.p.) injection of ketamine (100 mg/kg) and xylazine (6 mg/kg), and then 2×10^5^ MDA-MB-231-Luc2 cells suspended in 100 µl of PBS were injected into the left ventricle of each mouse as previously described [26]. Successful intra-cardiac injections were confirmed by immediate whole body bioluminescence imaging (showing systemic distribution of bioluminescence signal), and mice were randomly assigned to different experimental groups. BAPN intraperitoneal injections (Sigma-Aldrich; St. Louis, MO), at a dose of 100 mg/kg in 100 µl PBS, were initiated either 1 day prior, on the same day as, or 7 days after intracardiac tumor cell injection (experimental groups were abbreviated as ‘BAPN d−1’, ‘BAPN d0’, and ‘BAPN d7’). BAPN injections were performed daily until the end of the experiment. A control group of mice received daily i.p. injections with vehicle (PBS) only. Numbers of animals included into the analyses were n = 10 for the ‘no BAPN’ and for ‘BAPN d7’ groups, and n = 5 for the ‘BAPN d−1’ and for ‘BAPN d0’ groups. Development of metastases was monitored via bi-weekly bioluminescence imaging. Mice were sacrificed 21 d after the MDA-MB-231-Luc2 cell injections, and femora/tibiae were harvested for micro-computed tomography (μCT) and histology; in addition, internal organs were harvested for the *ex vivo* evaluation of bioluminescence so as to confirm the anatomical distribution of soft tissue metastases.

### Bioluminescence imaging

Bioluminescence imaging was performed as described previously [27], [28], [29]. Mice were administered *D*-luciferin (Gold Bio Technology; St. Louis, MO) at a dose of 150 mg/kg in PBS by i.p. injection, and anesthetized with 1.5–2% isofluorane for 10–12 min prior to imaging. Animals were then placed onto the warmed stage inside of the IVIS light-tight chamber and anesthesia was maintained with 1.5–2% isofluorane. For the image acquisition, the cooled charge coupled device (CCD) camera of Xenogen IVIS Lumina system (Caliper Life Sciences) was used; mice were imaged in both the dorsal and ventral positions. Imaging parameters were f/stop 1, bin 4, field of view 12.5 cm, and exposure times ranged from 20 s to 2 min, depending on the strength of the tumor-derived photon emission rates. Results were analyzed using Living Image 3.0 software (Caliper Life Sciences). Signal intensity was quantified as the total photons/s within a uniform region of interest positioned over specific tumor sites (as well as over the entire body) during the data post-processing, with any background bioluminescence subtracted out. For *ex vivo* imaging, organs of interest were dissected, placed into 24-well tissue culture plates along with 300 µg/ml *D*-luciferin in PBS, and imaged for 1–2 min using the IVIS system.

### Micro-computed tomography

Bone loss resulting from osteolytic metastases was assessed by μCT. Mice were sacrificed 21 d after intracardiac cell injection and bones were dissected and cleaned of soft tissues before fixing for at least 24 hrs in 10% neutral buffered formalin. For scanning, bones were first rinsed in PBS, and then placed into an airtight cylinder sample holder of a μCT scanner (μCT40, Scanco Medical, Brüttisellen, Switzerland). Regions of interest were selected following a scout view, and 400–800 serial tomographic images with a 10 µm increment were acquired at 55 kV and 145 µA, and a 200 ms integration time. The three-dimensional reconstructed images of the proximal tibia were analyzed (Image Processing Language, v5.05a). The 3D images included a region represented by 300 tomographic slices (3 mm) obtained immediately distal to the proximal tibial growth plate, and the raw images were Gaussian filtered to reduce noise (σ = 0.7, support of three voxels) and thresholded (255/1000) to extract mineralized bone tissue. Total bone volume was used as a measure of the magnitude of tumor-induced osteolysis.

### Histology

Following *ex vivo* bioluminescence imaging, selected organs were fixed in 10% neutral buffered formalin and long bones were fixed in 4% paraformaldehyde in phosphate buffer for 1 week followed by decalcification in 14% EDTA (with 14 consecutive changes) at 4°C. Tissues were then processed, embedded in paraffin, sectioned at 4 µm, and stained with hematoxylin and eosin.

### Statistical analyses

All data were plotted as mean±SEM. Statistical analysis was performed using ANOVA and Mann-Whitney *t*-test in GraphPad Prizm 4.0 Software (GraphPad Software Inc., San Diego, CA). Values of *P*<0.05 were considered statistically significant.

## Results

### Generation of luciferase-expressing MDA-MB-231 cells

**Figure 1:**
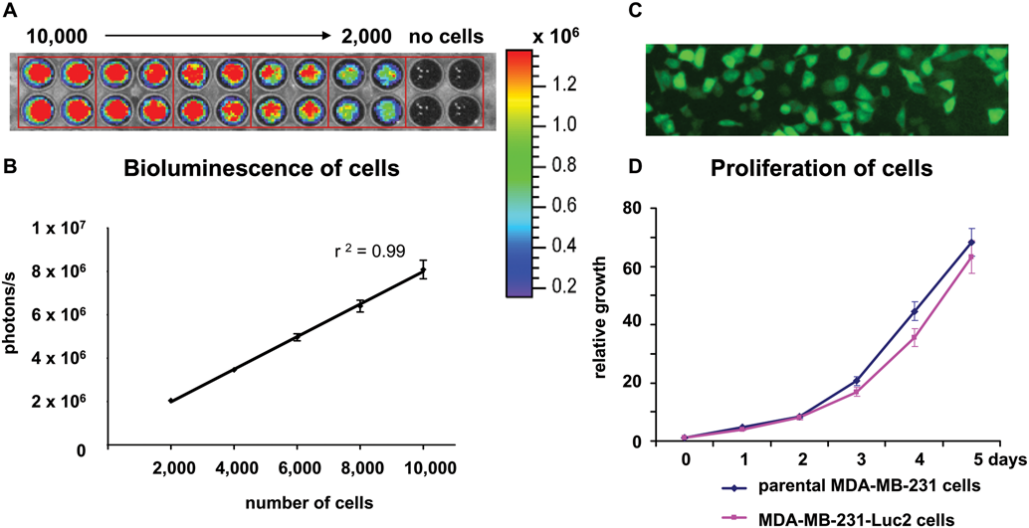
*In vitro* evaluation of MDA-MB-231-Luc2 cells. (A) Cells were plated in a 96-well plate in quadruplicate, ranging from 10,000 to 2,000 cell/well. Wells with media only (no cells) were included as control. *D*-luciferin substrate was added to each well and the plate was imaged to estimate the bioluminescence of MDA-MB-231-Luc2 cells. (B) Correlation between bioluminescence and cell number per well was plotted as mean photons/s/well±SEM. (C) Fluorescence microscopy, showing that MDA-MB-231-Luc2 cells expressed EGFP. (D) 1×10^3^ MDA-MB-231 parental and MDA-MB-231-Luc2 cells were seeded into quadruplicate wells of 96-well plates and their proliferation rates determined by MTT assay. Data was plotted as mean relative growth±SEM.

MDA-MB-231 cells were stably transfected with a plasmid encoding the EGFP-Luc2 fusion-protein as described in the ‘Materials and Methods’. FACS was then used to select the population of cells showing the highest EGFP signals. The MDA-MB-231-Luc2 line, composed of cells derived from the expansion and pooling of sixty individual clones, exhibited a strong correlation between cell number and bioluminescence signal intensity (r^2^ = 0.99) (**Fig. 1A, B**). The expression of luciferase, and EGFP (**Fig. 1C**) in MDA-MB-231-Luc2 cells was stable after the cells were cultured for several weeks in media lacking G418 selection (data not shown). After expansion, the pooled population was frozen-back in multiple aliquots, so that all subsequent xenografting experiments utilized the same pool of MDA-MB-231-Luc2 cells. The light emission for these cells ranged from 733 to 937 photons/s per cell (**Fig. 1B**), a rate that was several times higher than that of previously described MDA-MB-231-Luc cells [27]. The proliferation of the parental MDA-MB-231 and MDA-MB-231-Luc2 was similar (**Fig. 1D**), demonstrating that expression of the EGFP-Luc2 fusion-protein did not alter cell growth.

### Effect of BAPN treatment on development of MDA-MB-231-Luc2 metastasis and on whole body tumor burden

To evaluate the effect of LOX inhibition on the development of metastases, we introduced MDA-MB-231-Luc2 cells into the arterial circulation of NIH-III mice via intra-cardiac injection and then monitored the development of tumors at regular intervals using bioluminescence imaging. Mice were randomized into different experimental groups and daily BAPN treatments were initiated either the day before (d −1), the day of (3 hrs post-IC) (d 0), or 7 days (d 7) after MDA-MB-231-Luc2 intracardiac injection.

Initiation of treatment on d−1 was selected to try determine whether BAPN pretreatment might have an effect on early events, such as tumor cell adherence to the vascular endothelium and/or transmigration through the vessel wall; while BAPN given at the 3 hrs post-injection (d 0), a time when tumor cell transendothelial migration was either in-progress or complete [3], [30], [31], [32], was used to evaluate whether BAPN treatment might have an effect on the later stages of extravasation and/or on colonization of tumor cells in target tissues. These two BAPN administration times enabled us to obtain an idea as to whether BAPN might show a differential effect on the earliest stages of tumor cell-vessel wall interactions and extravasation versus the later stages of tissue colonization. In addition, to determine whether BAPN had any effect on the growth of established metastases, the chemical was given to mice at 7 days (d 7) post-injection, a time when bioluminescence signals from the metastases could be reliably measured. Mice from the control group were injected with PBS vehicle only.

**Figure 2:**
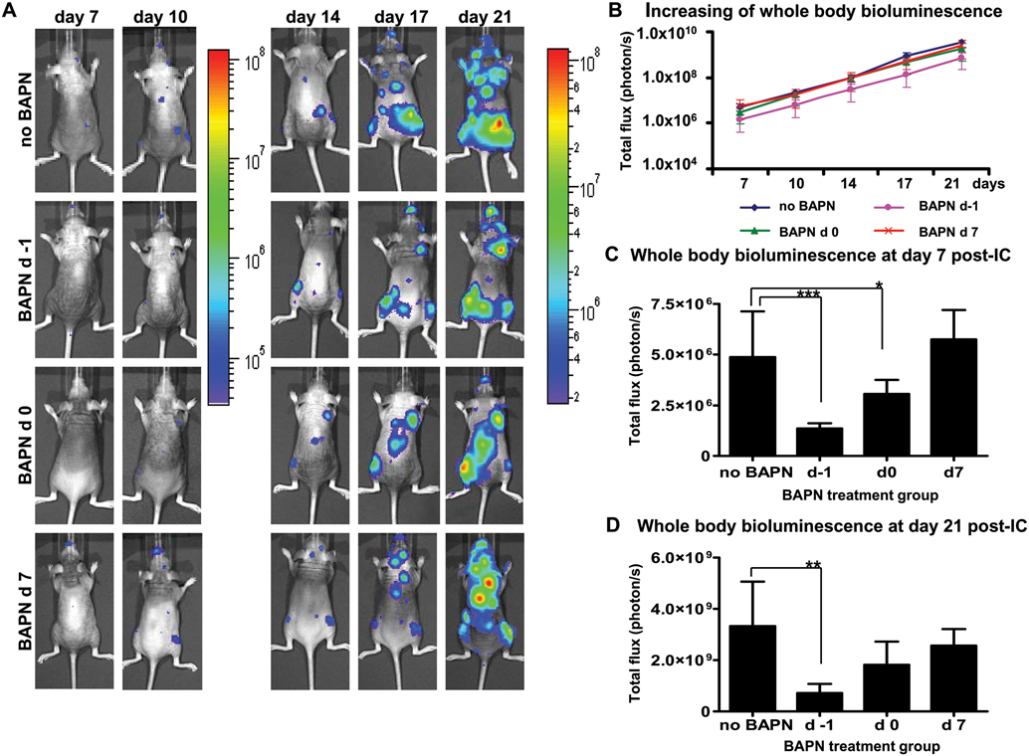
Growth of MDA-MB-231-Luc2 metastasis and the effects of BAPN treatment. Bioluminescence imaging was performed 2 times per week starting on day 7 after intracardiac injection of tumor cells. Representative dorsal images of control and BAPN-treated mice are shown from 7 to 21 d post-injection (A). Images for days 7–10, and days 14 to 21 are shown with different sensitivity color scale bars, reflecting the rapid growth rate of metastases. Scales are in photons/s/cm^2^. Whole body luminescence, a measure of tumor burden, for the different groups is shown as a function of time (B); note the need for a log scale owing to the rapid growth of the tumors. BAPN treatment was initiated at the indicated times and then continued daily thereafter until day 21. (C) Whole body bioluminescence at day 7 post-cell injection was quantified as mean photons/s±SEM. Whole body tumor bioluminescence at day 21 (D) was quantified as mean photons/s±SEM. For both (B) and (C), n = 10 for each of ‘no BAPN’ and for ‘BAPN d 7’ groups, and n = 5 for ‘BAPN d −1’ and for ‘BAPN d 0’ groups. Asterisks indicate statistical significance (^*^ P<0.05;^**^ P<0.01; ^***^ P<0.001).

Representative bioluminescence images of mice from all four experimental groups, taken at days 7, 10, 14, 17, and 21 post-intracardiac injection of MDA-MB-231-Luc2 cells, are displayed in **Figure 2A**. Quantification of whole-animal photon emission rates (**Fig. 2B**) allowed us to gauge relative tumor burden in the mice over time, and revealed that BAPN had a negative effect on the development of metastases, particularly when administered on d −1, but was ineffective when started on day 7 post-injection (**Fig. 2C**). Specifically, in the d −1 and d 0 groups, whole body bioluminescence at day 7 post-IC was reduced by 72% (P = 0.0001) and 37% (P = 0.0240), respectively, as compared to the ‘no BAPN’ animals (**Fig. 2C**). Similarly, at day 21 post-injection, whole body tumor burden was decreased by 78% (P = 0.0018) and 45%, respectively, in these two groups of mice, as compared to the control group (**Fig. 2D**). BAPN administration after metastases had already formed (d 7) again had no significant effect on tumor growth (**Fig. 2D**).

### BAPN reduces the number of MDA-MB-231-Luc2 metastases

**Figure 3:**
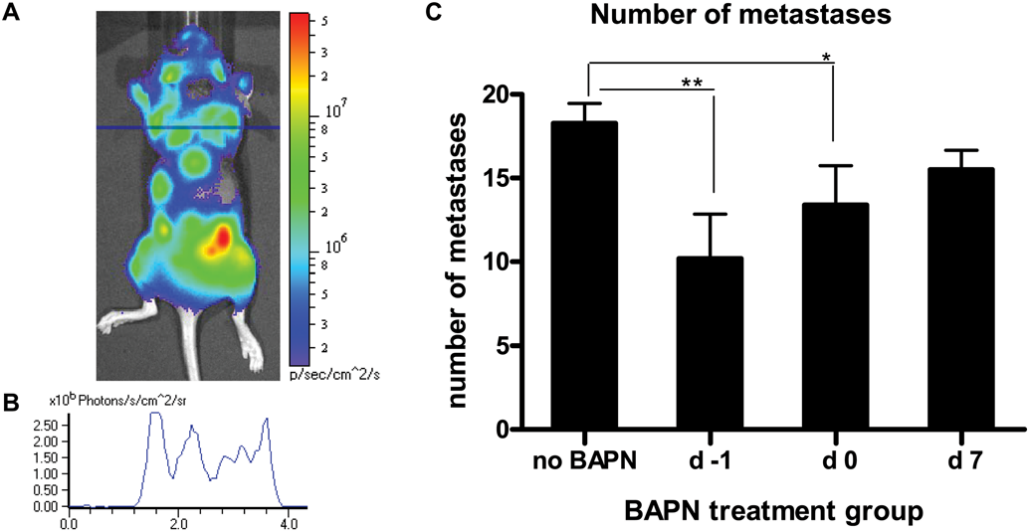
Effect of BAPN administration on the number of metastases. Metastases were quantified by moving a line for measuring photon emissions over the entire mouse image (A), and using this to count the number of bioluminescent peaks in the whole animal profile (B). Dorsal, ventral, and lateral images taken at day 21 after cell injection were evaluated for mice that received BAPN starting at the indicated time points, and continued daily thereafter until day 21, as well as for control (vehicle only) mice. Total number of metastases per mouse are shown as the mean±SEM (C) (n = 10 for each of ‘no BAPN’ and for ‘BAPN d 7’ groups, and n = 5 for ‘BAPN d −1’ and for ‘BAPN d 0’ groups). Asterisks indicate statistical significance (^*^ P<0.05; ^**^ P<0.01).

There is evidence that only a small proportion of extravasated cells go on to form metastases, while the bulk of the tumor cells either remain dormant within their metastatic sites or undergo apoptosis after a few cell divisions [3], [33], [34]. To gain insight into whether BAPN treatment had an effect on the number of metastases we examined bioluminescence line profiles obtained from dorsal, lateral, and ventral images of mice (**Fig. 3A, B**). This estimate revealed that when BAPN treatment was initiated either on d −1 or d 0, the average number of metastatic sites per mouse decreased by 44% (P = 0.0028), and 27% (P = 0.0284), respectively, as compared to the ‘no BAPN’ controls (**Fig. 3C**). Administration of BAPN after metastases had developed did not reveal any significant reduction of number of metastases per mouse (**Fig. 3C**).

### Anatomic distribution of MDA-MB-231-Luc2 metastases in BAPN-treated mice

**Figure 4:**
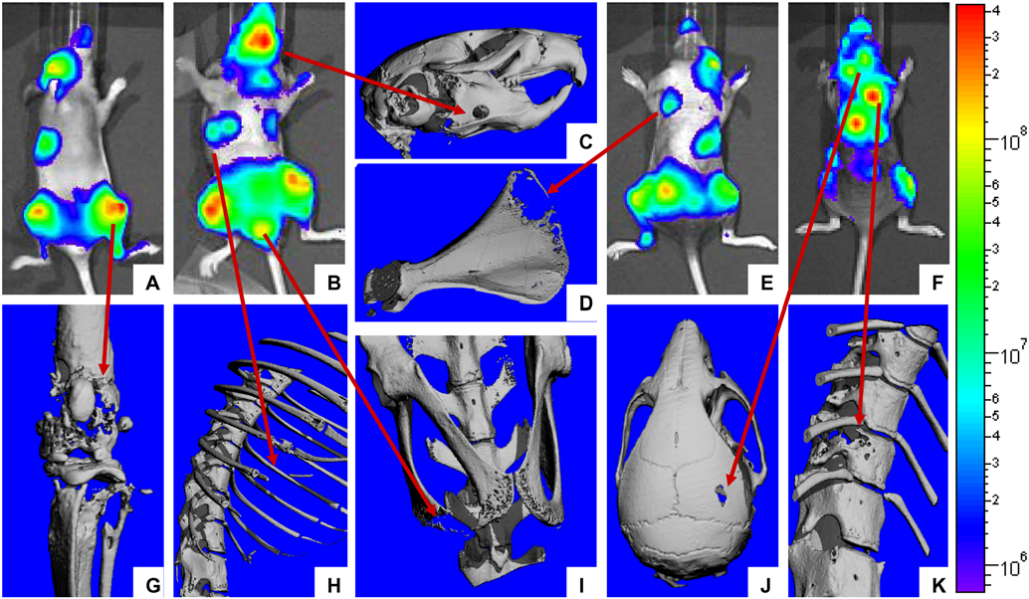
Correlation between tumor bioluminescence and osteolytic lesions. Representative bioluminescence images (A, B, E, F) and corresponding 3D μCT reconstructions for some of the most common skeletal sites of bone metastases in NIH-III mice following intracardiac injection of MDA-MB-231-Luc2 cells: jaw (C), scapula (D), knee (G), ribs (H), pubis/ischium (I) parietal bone of the skull (J), and thoracic spine (K). Scale bar on the right of images represents intensity of bioluminescence in photons/s/cm^2^.

Since breast cancer cells exhibit tropisms for specific tissues [4], [26], [35], we evaluated whether BAPN treatment might alter tissue-specific pattern of metastasis. The pattern of MDA-MB-231-Luc2 metastases was thus established via (i) bioluminescence imaging of the mice, (ii) determination of isolated organ bioluminescence *ex vivo*, and (iii) μCT imaging of the skeleton. This analysis, carried out for the ‘no BAPN’ control group demonstrated that MDA-MB-231-Luc2 cells were able to metastasize to a variety of different soft tissue and skeletal sites (**Table 1**), with bone metastases exhibiting a strong predilection for specific skeletal sites, such as, the scapulae, distal femora/proximal tibiae, ribs, pelvis, vertebral bodies, and mandible) (**Fig. 4** **and** **Table 1**). Interestingly, the frequencies of tissue-specific metastases in the mice were reminiscent of those observed in individuals with metastatic breast cancer [36], [37]. We found that BAPN administration did not significantly alter the anatomical distribution of metastases (**Table 1**).

**Table 1:** Frequency of metastases at various anatomical sites following the intracardiac injection of MDA-MB-231-luc2 cells in control, and BAPN-treated mice.

Site of metastases	no BAPN, % (frequency)	BAPN d 7, % (frequency)	BAPN d 0, % (frequency)	BAPN d −1, % (frequency)
**Soft tissue sites:**				
Lungs	100 (10/10)	90 (9/10)	100 (4/4)	100 (5/5)
Heart	80 (8/10)	100 (10/10)	100 (4/4)	100 (5/5)
Peritoneum*	70 (7/10)	30 (3/10)	25 (1/4)	0 (0/5)
Pancreas	40 (4/10)	40 (4/10)	25 (1/4)	0 (0/5)
cervical lymph nodes	30 (3/10)	40 (4/10)	25 (1/4)	40 (2/5)
adrenal gland	20 (4/20)	30 (6/20)	25 (2/8)	10 (1/10)
Ovaries	5 (1/20)	25 (5/20)	25 (2/8)	30 (3/10)
Liver	0 (0/10)	0 (0/10)	20 (1/4)	0 (0/5)
Stomach	0 (0/10)	10 (1/10)	0 (0/4)	0 (0/5)
Bladder	0 (0/10)	10 (1/10)	0 (0/4)	0 (0/5)
**Skeletal sites:**				
knee (tibia and/or femur)	100 (20/20)	100 (20/20)	100 (10/10)	70 (7/10)
Ribs	100 (10/10)	90 (9/10)	100 (5/5)	80 (4/5)
Mandible	90 (9/10)	80 (8/10)	80 (4/5)	80 (4/5)
vertebrae**	90 (9/10)	80 (8/10)	100 (5/5)	20 (1/5)**
proximal femur	50 (10/20)	35 (7/20)	30 (3/10)	30 (3/10)
iliac crest	40 (8/20)	60 (12/20)	40 (4/10)	40 (4/10)
Scapulae	45 (9/20)	50 (10/20)	50 (5/10)	30 (3/10)
parietal bones of the skull	30 (3/10)	40 (4/10)	40 (2/5)	20 (1/5)
proximal humerus/shoulder	15 (3/10)	10 (2/20)	10 (1/10)	10 (1/10)
ankle/paw	10 (4/40)	18 (7/40)	10 (2/20)	0 (0/20)
pubic symphysis	10 (1/10)	50 (5/10)	0 (0/4)	0 (0/5)

* P<0.05.

** P<0.01.

### BAPN pre-treatment inhibits the development of MDA-MB-231-Luc2 bone metastases

**Figure 5:**
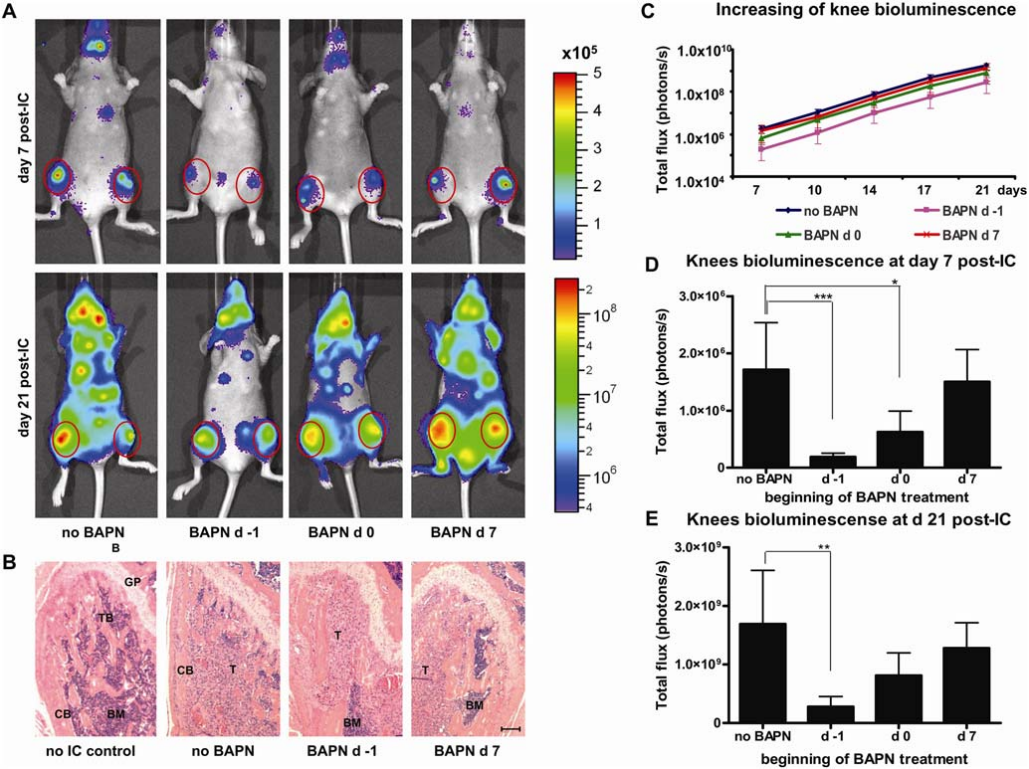
Development of MDA-MB-231-Luc2 metastases in the knees of mice receiving BAPN as compared to controls. Representative ventral bioluminescence images of control and BAPN-treated mice are shown for the days 7 and 21 following injection of cells (A) with circles over the knees illustrating the placement of the regions of interest (ROI). (B) H&E stained sections showing the growth plate (GP), cortical bone (CB), trabecular bone (TB), bone marrow (BM), and tumor area (T) of representative right femurs; the scale bar is 50 µm. (C) Knee bioluminescence increases over time; at day 7 (D), and day 21 (E) after cell injection. Signals were quantified from dorsal and ventral knee ROI and graphed as mean photons/s±SEM (n = 10 for each of ‘no BAPN’ and for ‘BAPN d 7’ groups, and n = 5 for ‘BAPN d −1’ and for ‘BAPN d 0’ groups). Asterisks indicate statistical significance (^*^ P<0.05; ^**^ P<0.01; ^***^ P<0.001).

Bone is the most common site of metastasis in human breast cancer [37], [38], and it was also the preferred site of MDA-MB-231-Luc2 metastases, with one or both knees (distal femur and/or proximal tibia) being involved 100% of the time (**Table 1**). To monitor the development and progress of skeletal metastases at a given site in response to BAPN, bioluminescence measurements, and histological analysis were performed on the involved knees (**Fig. 5A,B**), and photon emission rates were quantified as a function of time (**Fig. 5C**). As with the whole body analysis, when BAPN treatment was initiated early, knee metastases were significantly reduced. Thus, in mice where BAPN treatment was started on d −1, and on d 0, the average knee bioluminescence (taken at day 7) was reduced by 89% (P = 0.0001), and 63% (P = 0.0424), respectively, as compared to vehicle-injected mice (**Fig. 5D**). At 21 d post-injection the average knee bioluminescence was still reduced in these groups of mice by 83% (P = 0.0271) for the ‘BAPN d −1’group, and by 52% for the ‘BAPN d 0’ group, as compared to controls (**Fig. 5E**). BAPN treatment initiated after metastases had formed (at day 7 post-injection) did not significantly reduce tumor bioluminescence. These results suggest that BAPN pre-treatment reduced the number of circulating tumor cells able to colonize the proximal tibia in the early hours post-intracardiac injection. Thus, while not eliminating knee metastases altogether, BAPN pre-treatment significantly reduced tumor growth at this site (**Fig. 5D,E**) perhaps as a consequence of decreased intramedullary colonization by MDA-MB-231 cells.

**Figure 6:**
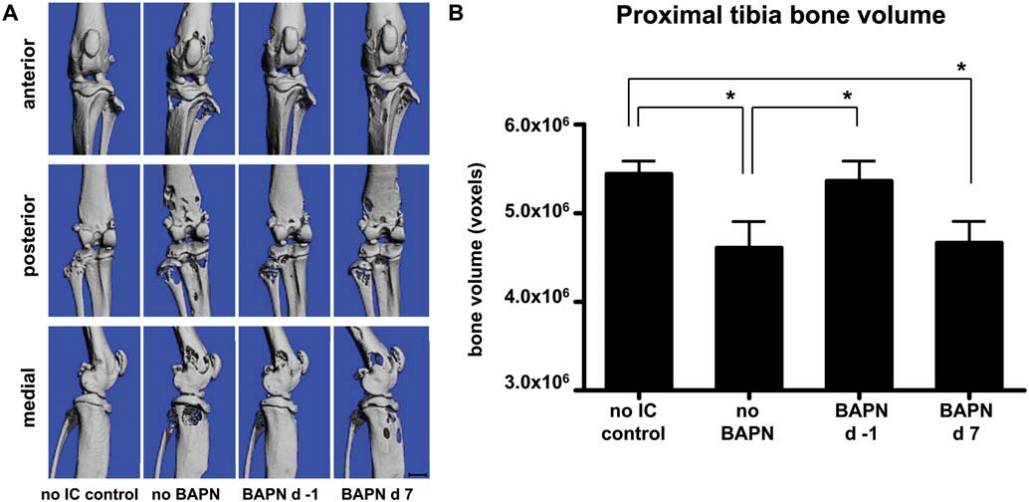
Evaluation of knee osteolysis by μCT. Mice undergoing BAPN treatment and their controls were sacrificed at day 21 after intracardiac injection of MDA-MB-231-Luc2 cells, and their left hind limbs were examined by μCT. Hind limbs from age-matched non-tumor cell-injected mice were used as controls. Anterior, posterior, and medial μCT images of representative knee joints from the different groups are shown (A). Bone volumes were determined from measurements of three hundred 10 µm slices taken just distal to the proximal tibial growth plate and graphed as mean bone volume±SEM (B) (n = 8 for ‘no IC’, that is, control mice that were not injected with tumor cells; n = 9 for ‘no BAPN’; n = 10 for ‘BAPN d 7’; and n = 5 for ‘BAPN d −1’ groups). Asterisk indicates a statistical significance of P<0.05.

To determine whether BAPN would show an effect on tumor-induced osteolysis, quantitative μCT analysis was employed (**Fig. 6A**). The knees of control mice injected with MDA-MB-231-Luc2 cells lost about 15% of proximal tibial bone volume by day 21 (P = 0.0296), as compared to controls lacking tumors (**Fig. 6B**). A similar degree of bone loss was seen in mice where BAPN treatment was not started until d 7 post-injection (P = 0.0112). Thus, BAPN did not appreciably alter the degree of osteolysis caused by established tumors. In contrast, when BAPN was administered on d −1, tibial tumor size was smaller (as shown by the reduced photon emission rates)(**Fig. 5D, E**), and hence, bone volume was preserved and not significantly different from that of age-matched mice lacking tumors (**Fig. 6B**).

## Discussion

Non-invasive bioluminescence imaging of luciferase-expressing tumor cells is an excellent method for following tumors *in vivo*, providing not only an efficient way to quantify tumor growth over time, but also a means with which to identify both the numbers and anatomical sites of systemic metastases [39], [40].

The vector encoding a EGFP-Luc2 fusion-protein enabled us to use FACS to select for MDA-MB-231 transfectants with light emission rates exceeding those of previously described breast cancer cell lines. Thus, bioluminescence of our MDA-MB-231-Luc2 cells, in the range of 730–930 photons/s/cell (**Fig. 1A, B**), was higher than the 130–210 photons/s/cell obtained for individual clones of MDA-MB-231-Luc [27], or the 37–51 photons/s/cell reported for MDA-MB-435-luc cells [40]. Luciferase expression in our model allowed us to reliably quantify tumor burden in mice as early as 7 d after intra-cardiac injection of MDA-MB-231-Luc2 cells.

We used the MDA-MB-231-Luc2 xenograft system to demonstrate that the LOX inhibitor, β-aminopropionitrile, was able to decrease tissue colonization by MDA-MB-231-Luc2 cells following their intra-cardiac administration. Thus, BAPN pretreatment (day −1) significantly reduced not only the number of metastases that developed but also the whole animal tumor burden. The former result suggested that the numbers of cells ‘seeding’ a given tissue site had been reduced by BAPN pre-treatment. When BAPN initiated 3 hrs after tumor cell injection, there was less of an inhibition on metastasis development, suggesting that this agent was most effective when it was able to act on tumor cells and/or host tissues during the initial phase of the extravasation process. Although BAPN pre-treatment did not eliminate metastases altogether, it should be noted that in our experimental protocol, supra-‘physiological’ numbers of highly malignant tumor cells were being introduced into the circulation. It is therefore plausible that the metastasis-inhibiting effect of BAPN might prove even more complete in ‘real life’ scenarios where circulating tumor cell concentrations are thought to be exceedingly low. Although it is conceivable that BAPN can target other cellular molecules in addition to LOX, our findings suggest that LOX inhibition was responsible for the diminished tissue colonization by the circulating MDA-MB-231 cells.

Newly extravasated tumor cells are thought to remain quiescent during the first few days after intra-cardiac injection, only forming small metastatic foci (with less than 10 cells per focus) by the end of the first week [33], [41]. Observations using intra-vital microscopy have revealed that most of tumor cells introduced into the blood stream by intracardiac or tail vein injection adhere to the blood vessel endothelium during the first 5 min to 1 hr post-injection, extend their processes between the gaps in the endothelium and undergo trans-endothelium migration 30 min to 3 hrs later, and then complete their extravasation by 3–4 hrs post-injection [30], [42], [43]. Following the extravasation, tumor cells adhere to sub-endothelial matrix, and begin to remodel the surrounding tissue stroma allowing the subsequent colonization and growth of metastases [44]. Thus, the reduction of whole body tumor burden in mice pre-treated (day −1) with BAPN was likely a result of LOX inhibition during the initial steps of tumor cell interaction with the blood vessel epithelium; while the effect of BAPN in the ‘day 0’ group may have been due LOX inhibition during the later stages of extravasation and initial tissue colonization.

In blood vessels LOX is expressed both the endothelium and the vascular smooth muscle cells [14], [45], [46], [47]; LOX expression is also upregulated in invasive breast cancer cells [22], [23]. During catalytic deamination, LOX generates hydrogen peroxide, a species that can act as a chemoattractant for vascular smooth muscle cells [18] and breast cancer cells [20], and that also activates FAK and Src [20]; in this way, LOX may be able to indirectly modulate cell adhesion and motility [17], [48]. In addition, *in vitro* studies have shown that exposure of endothelial cells to hydrogen peroxide increases the permeability of this barrier [49], [50], [51], [52], [53]. Perhaps consistent with their ability to regulate blood vessel permeability, inhibition of FAK or Src signal transduction reduces tumor cell extravasation [43], [54]. Thus, a number of lines of research suggest a role for the FAK and Src kinases in regulation of vascular permeability during tumor cell extravasation, with LOX playing a regulatory role in this process. Our results suggest that LOX inhibition, perhaps by diminishing FAK activity [20], is able to suppress tumor cell-endothelial adhesion and/or extravasation. This might be of potential therapeutic relevance at times when elevated numbers of tumor cells are present in the circulation, such as immediately after breast cancer surgery [55], [56].

LOX up-regulation has been correlated with a worse prognosis breast cancer, thus, in addition to its induction during hypoxia [16], it has been proposed that LOX might be required for the growth of already established metastases [57]. Our results, however, show that once metastases have developed, BAPN administration had no appreciable impact on tumor growth. This was consistent with the findings of Erler and colleagues who showed that while BAPN was able to significantly reduce the formation of metastases at distant sites, it had no effect on the growth rate of MDA-MB-231 tumors at the orthotopic implantation site [16].

An advantage of the intracardiac route to study metastatic behavior of tumor cells lies in the high frequency with which metastases in multiple skeletal and soft tissue sites are obtained in this model [58]. MDA-MB-231-Luc2 cells formed metastases with frequencies and tissue- and site-specific patterns similar to those observed in individuals with metastatic breast cancer [36], [37]. Since catalytically active LOX, whose levels could plausibly differ between vascular beds, may be able to act as a chemoattractant and a modulator of adhesion and the invasiveness of tumor cells [17], [20], we investigated whether BAPN administration would alter the anatomical pattern of metastases. Although BAPN pre-treatment significantly reduced the total number of tumor deposits, it failed to significantly alter the anatomical pattern of metastases.

Evaluation of the effect of BAPN on the development of skeletal metastases was of special interest as bones were the most common site of metastasis observed in our mice after the intra-cardiac injections of MDA-MB-231-Luc2 cells, and they also represent the most common site of metastasis in breast cancer [37], [38]. Our findings demonstrated that BAPN was able to reduce the development of bone metastases. Thus, in mice where the BAPN treatment was initiated on day −1, tumor burden in knees was reduced about 9–fold at day 7 post-injection as compared to controls. Our observations at earlier time points (data not shown), that were in agreement with previous reports [41] (and our own unpublished data), demonstrating that breast cancer cells tend to colonize mouse bones in the region of the primary spongiosa immediately beneath the epiphyseal growth plate. Perhaps LOX activity [18], [20], [59], in combination with other factors released by osteoblasts, hypertrophic chondrocytes, and/or stromal cells promotes tumor cell chemo-attraction, motility, proliferation, and survival at these skeletal sites.

In summary, the LOX inhibitor, BAPN, was able to significantly decrease the frequency of metastases in animals were challenged by the introduction of large number of breast cancer cells directly into the arterial circulation. Our results suggest that LOX plays an important role in extravasation and/or tissue colonization by tumor cells, lending support to the idea that LOX inhibition might be useful in metastasis prevention.
